# The limited value of routine radiographs in the follow-up treatment of proximal humerus fractures

**DOI:** 10.1007/s00068-026-03333-y

**Published:** 2026-09-23

**Authors:** A. A. Elwakel, G. J. A. Willinge, J. C. Goslings, B. A. Twigt

**Affiliations:** 1 OLVG Hospital, Amsterdam, The Netherlands; 2 Amsterdam UMC, Amsterdam, The Netherlands

**Keywords:** Proximal humerus fracture, Radiography, Follow-up, Conservative treatment, Fracture displacement, Trauma surgery

## Abstract

**Purpose:**

Proximal humerus fractures (PHFs) are common injuries in adults and occur particularly often in older patients. In our hospital, patients are routinely scheduled for follow-up radiographs at approximately two and six to eight weeks after injury, although the management yield of this practice is uncertain.

**Methods:**

We performed a retrospective cohort study of consecutive adults (≥ 18 years) presenting with PHFs to the OLVG emergency department (ED) between January and April 2022. Patients with multiple fractures, pathological fractures, fractures initially treated or followed up at another hospital, and fractures other than proximal humerus fractures were excluded. The primary outcome was a documented treatment-plan change during the observed outpatient treatment period, including conversion from conservative to operative treatment, cancellation of planned surgery, or additional surgery after initial operative treatment. The primary outcome proportion was reported with a two-sided exact 95% confidence interval. Descriptive variables included fracture classification, comminution, imaging use, follow-up timing, secondary displacement, and treatment duration.

**Results:**

One hundred patients were included (mean age 60.4 ± 18.0 years; 60% female). Most fractures were one-part (63%) or two-part (33%), 45% showed comminution, and 87% of patients were treated conservatively. The observed treatment-plan change rate was 0/100 (0%; exact 95% confidence interval, 0.0–3.6%). Among the 87 patients initially treated conservatively, none converted to operative treatment. Secondary displacement occurred in nine patients, including one radiographic non-union and one malunion, but no treatment-plan change was documented in these patients. No revision surgery was performed. Patients underwent a mean of 3.0 radiographs, including the initial ED radiograph.

**Conclusion:**

In this cohort, routine follow-up radiographs had a low observed management yield: none was associated with a documented treatment-plan change. Because the upper 95% confidence limit was 3.6% and management reflected practice within a single hospital organisation, these data do not establish the safety of omitting radiographs. They support larger multicentre evaluation of clinically triggered rather than universally scheduled follow-up imaging.

## Introduction

Proximal humerus fractures (PHFs) are common fractures in adults and occur particularly often in older women [1–4]. Although the burden of PHFs is highest in older adults, these fractures also occur in younger adults, who may have different functional demands and treatment considerations. In clinical practice, follow-up protocols are generally applied to the adult PHF population as a whole, not only to older patients. PHFs account for approximately 5–6% of all fractures, with the majority occurring in females aged 65 years and older [3, 4]. In individuals over 65, PHFs represent 10% of all fractures and rank as the third most common type of osteoporotic fracture [5, 6]. The management of patients with PHFs depends on several factors, including the type and degree of fracture displacement, patient age, and associated comorbidities. Most patients are treated conservatively using immobilization with a sling and subsequent physical therapy [7]. In our hospital, patients are usually followed up with radiographic imaging two and six to eight weeks after their initial assessment, with some patients receiving additional follow-up imaging. However, complications identified on follow-up imaging are rare [8], and even when radiologic findings are present, the treatment plan is seldom altered to a surgical intervention [9].

This study aimed to describe the clinical course and fracture characteristics of adults aged ≥ 18 years with a proximal humerus fracture and to quantify the observed management yield of routine follow-up radiographs during outpatient follow-up.

We hypothesized that the observed management yield of routine follow-up radiographs would be low and that these data could inform the design of a larger study evaluating selective imaging.

## Patients and methods

### Inclusion and exclusion

For this retrospective cohort study, we screened consecutive patients who presented to the Emergency Department (ED) and were suspected of having a PHF from January 2022 to April 2022. We included all consecutive eligible patients within this defined four-month period, during which the institutional follow-up routine was unchanged and electronic records were complete. The sampling frame was pragmatic rather than based on a formal sample-size calculation; the precision of the primary outcome was therefore quantified using an exact 95% confidence interval. Exclusion criteria were: initial presentation or follow-up in another hospital, fractures other than proximal humerus fractures including midshaft humeral fractures, no fracture on imaging, pathological fracture, polytrauma or multiple fractures, and loss to follow-up before the first scheduled follow-up assessment.

### Primary Outcome

The primary outcome was the proportion of patients with a documented treatment-plan change during the observed treatment period, defined as the period from the initial ED visit until the final outpatient clinic visit. All available radiographs and outpatient clinic notes during this period were reviewed. This endpoint captured treatment decisions made in routine practice within the study institution; it was not designed to estimate how surgeons outside this institution might have managed the same radiographic findings.

A treatment-plan change was defined as a change from the treatment strategy chosen after the initial assessment. In patients initially managed conservatively, this referred to conversion to operative treatment during follow-up. In patients initially scheduled for surgery, this referred to cancellation of the planned operation in favour of continued conservative treatment before surgery. For patients who underwent surgery, we assessed whether additional surgery occurred during the observed follow-up period after follow-up imaging. Fractures were classified according to the Neer classification [10].

### Descriptive variables and statistical analysis

We collected descriptive data on follow-up timing and findings, imaging use, treatment duration, fracture classification, comminution, and secondary displacement. Secondary displacement was described without inferential risk-factor analysis because the number of events was small.

Continuous variables were summarized using means and standard deviations or medians and interquartile ranges, as appropriate. Categorical variables were presented as frequencies and percentages. The primary outcome was reported as a proportion with a two-sided exact 95% confidence interval calculated using the Clopper–Pearson method. No hypothesis test was performed for the primary outcome.

## Results

### Demographic and baseline characteristics

**Fig. 1:**
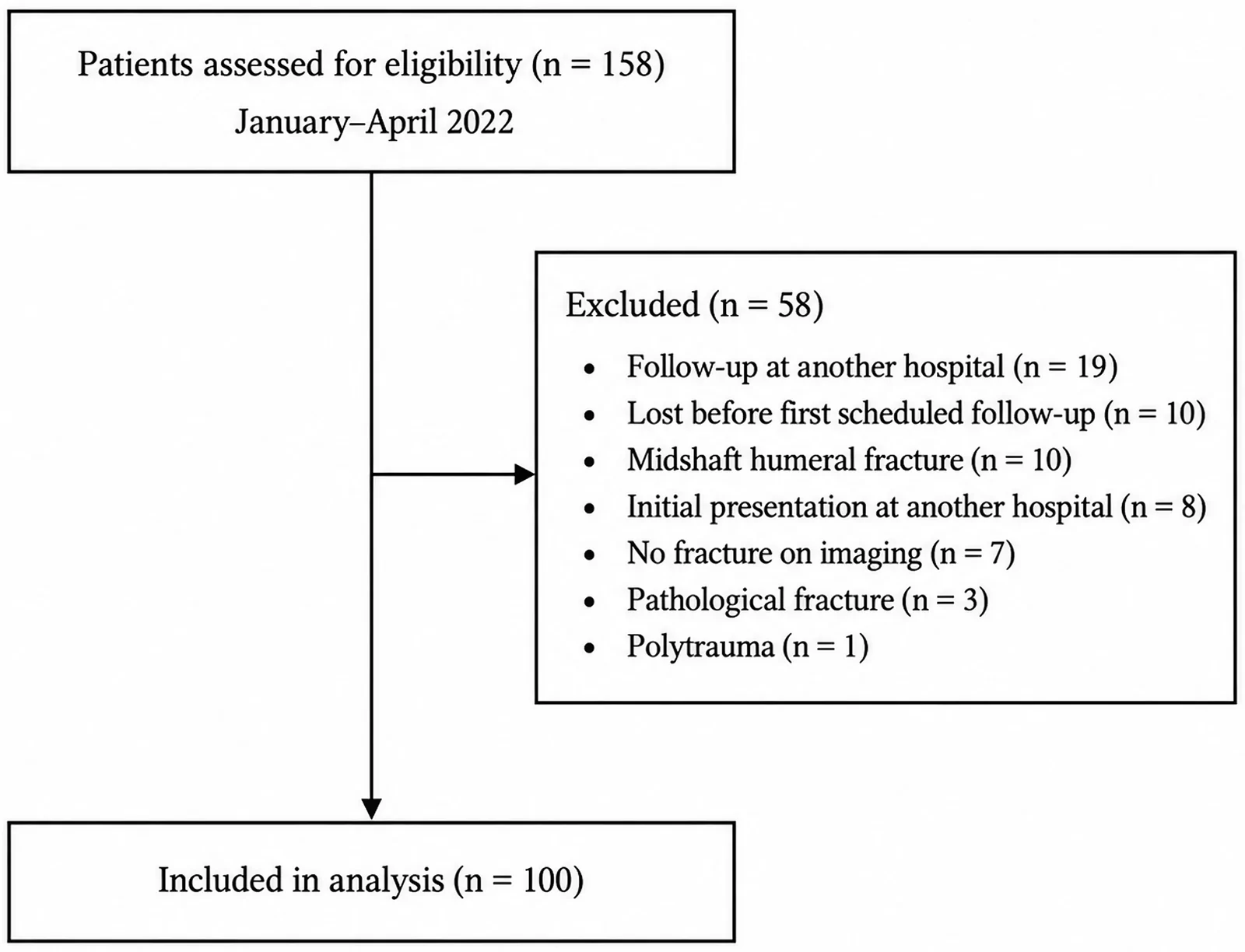
Flowchart of patient inclusion and exclusion

**Fig. 2:**
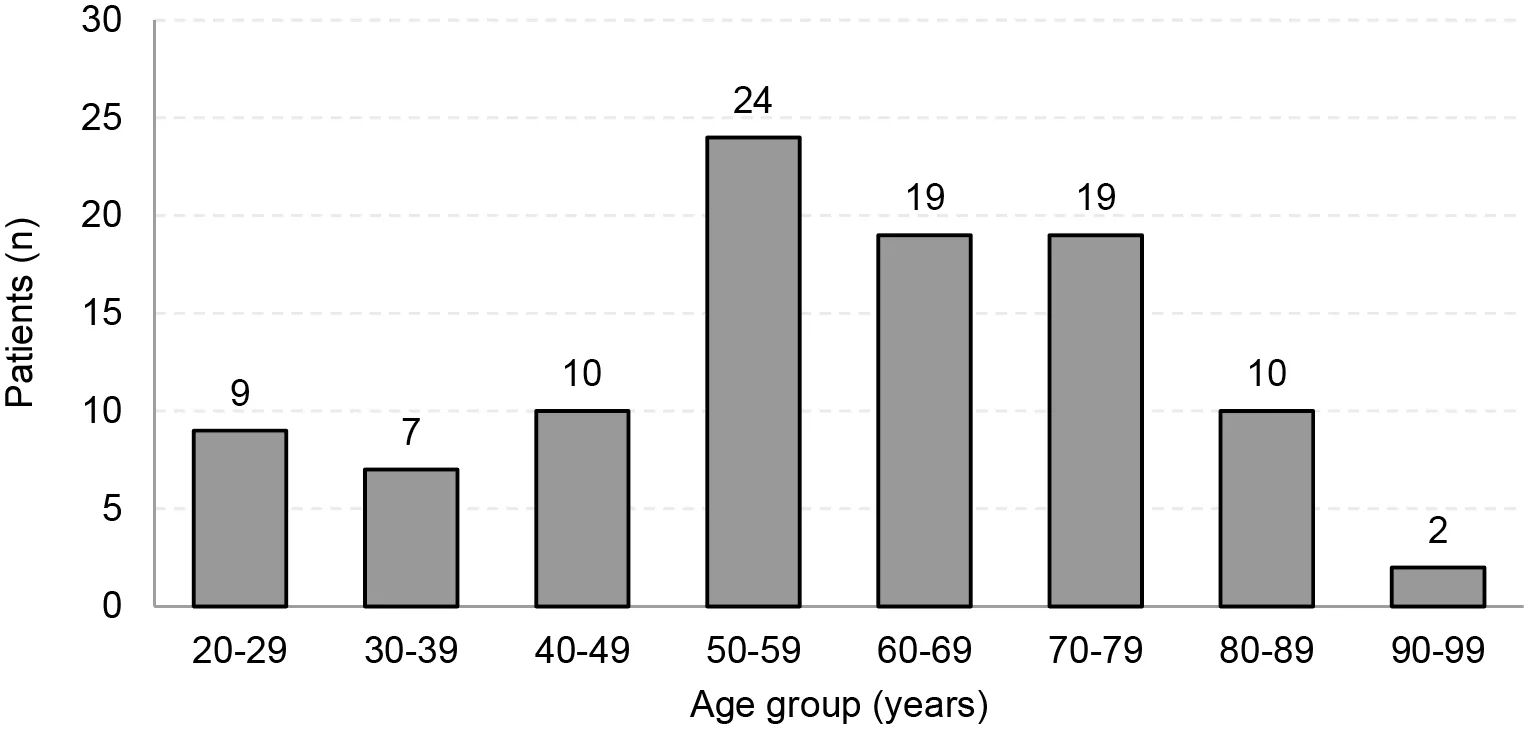
Age distribution of the study cohort

In total, 158 patients were assessed for eligibility and 100 patients were included in the study (Fig. 1). Fifty-eight patients were excluded because follow-up was performed in another hospital (*n* = 19), they were lost to follow-up (*n* = 10), the fracture was not a proximal humerus fracture including midshaft humeral fractures (*n* = 10), initial presentation was in another hospital (*n* = 8), no fracture was present on imaging (*n* = 7), the fracture was pathological (*n* = 3), or the patient had polytrauma (*n* = 1). All patient characteristics are shown in Table 1. The mean age of the study population was 60.36 years (SD = 17.96) (Fig. 2). Of the participants, 40% were male and 60% were female. Smoking was documented in 23% of patients, abnormal alcohol consumption (defined as consuming more than 7 glasses per week [11]) in 18%, and drug use, defined as the non-medical consumption of psychoactive substances such as cannabis, cocaine, amphetamines, or opioids, in 12% (Table 1). Comorbidities were documented in 41% of the cases, with hypertension being the most common (18%), followed by cerebrovascular diseases (11%), and diabetes mellitus (11%), as shown in Table 2.

**Table 1:** Patient and fracture characteristics

Characteristics		Value
Age, years (mean ± SD)		60.36 ± 17.96
Female sex, *n* (%)		60 (60%)
Comorbidities, n (%)		41 (41%)
Smoking, n (%)		23 (23%)
Abnormal alcohol consumption, n (%)		18 (18%)
Drug use, n (%)		12 (12%)
Fracture configuration, n (%)	One-part	63 (63%)
	Two-part	33 (33%)
	Three-part	2 (2%)
	Four-part	2 (2%)
Comminution, n (%)		45 (45%)

**Table 2:** Comorbidities in the study cohort

Comorbidity	Percentage
Hypertension	18%
Cerebrovascular disease	11%
Diabetes mellitus	11%
Cardiovascular disease	10%
COPD	7%
Psychiatric disease	6%
Other	6%

### Primary outcome

Of the 100 included patients, no patient underwent a documented treatment-plan change following routine follow-up radiographs during the observed treatment period. Among the 87 patients initially managed conservatively, none converted to operative treatment. No operated patient underwent additional surgery based on follow-up imaging. Of the 13 operated patients, 10 had a two-part PHF with severe dislocation or angulation, and three had either a three- or four-part PHF.

Nine patients demonstrated secondary displacement on the two-week radiograph. One subsequently had radiographic non-union and one had malunion. No treatment-plan change was documented in these nine patients; clinic notes recorded continuation of nonoperative management after consideration of symptoms, function, patient factors, and patient preference.

### Descriptive follow-up and imaging findings

#### Secondary fracture displacement

Nine patients showed secondary displacement on their two-week radiographs. Secondary displacement was evaluated descriptively because the number of events was small.

#### Fracture classification and characteristics

Of all fractures, 63% were one-part, 33% were two-part, 2% were three-part, and 2% were four-part. Of the four patients with a three- or four-part PHF, three underwent an operation and one was treated conservatively. Of the 33 patients with a two-part PHF, 10 underwent surgery. Comminution was present in 45%.

#### Follow-up timing and findings

**Fig. 3:**
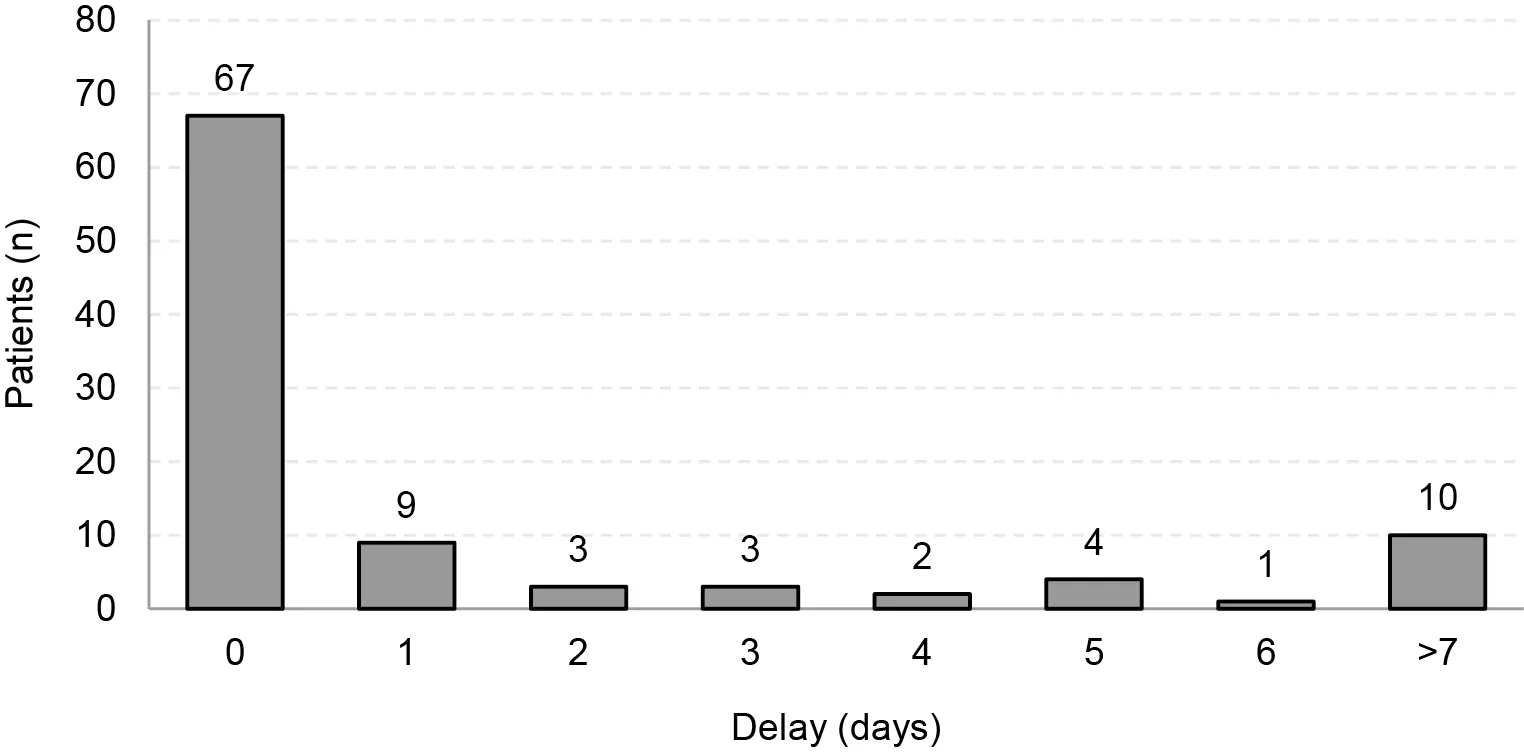
Delay between trauma onset and presentation to the emergency department

**Fig. 4:**
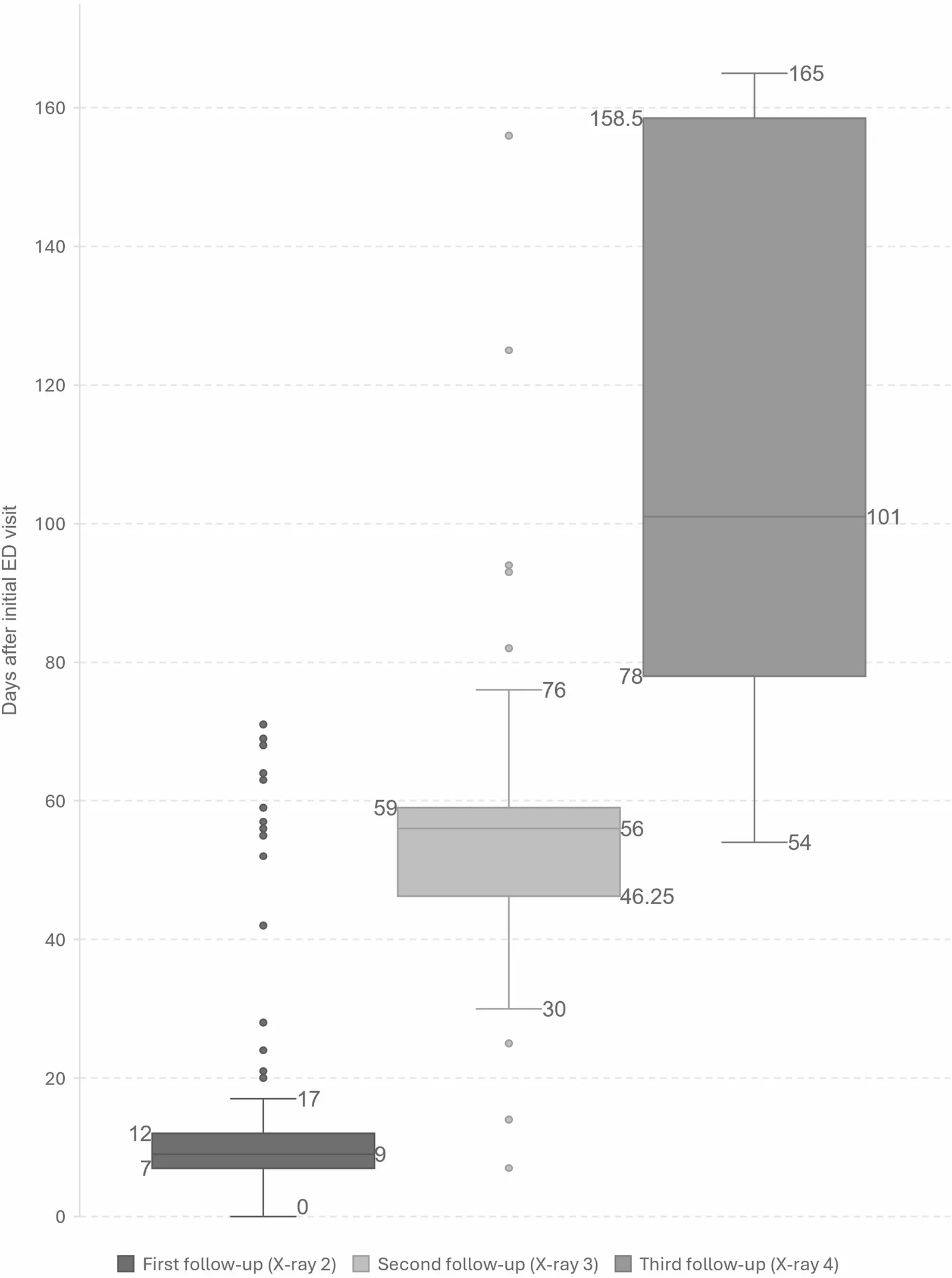
Follow-up intervals after the initial emergency department visit

The median interval between trauma and the initial ED visit was 0 days (IQR, 1; range, 0–34 days) (Fig. 3). The first follow-up assessment occurred after a median of 9 days (IQR, 5; range, 0–71 days), and the second after a median of 56 days (IQR, 12.75; observed range, 7–156 days) (Fig. 4). Four patients did not undergo a follow-up radiograph. One underwent CT instead, whereas the other three had documented functional improvement and no additional radiographic imaging.

**Fig. 5:**
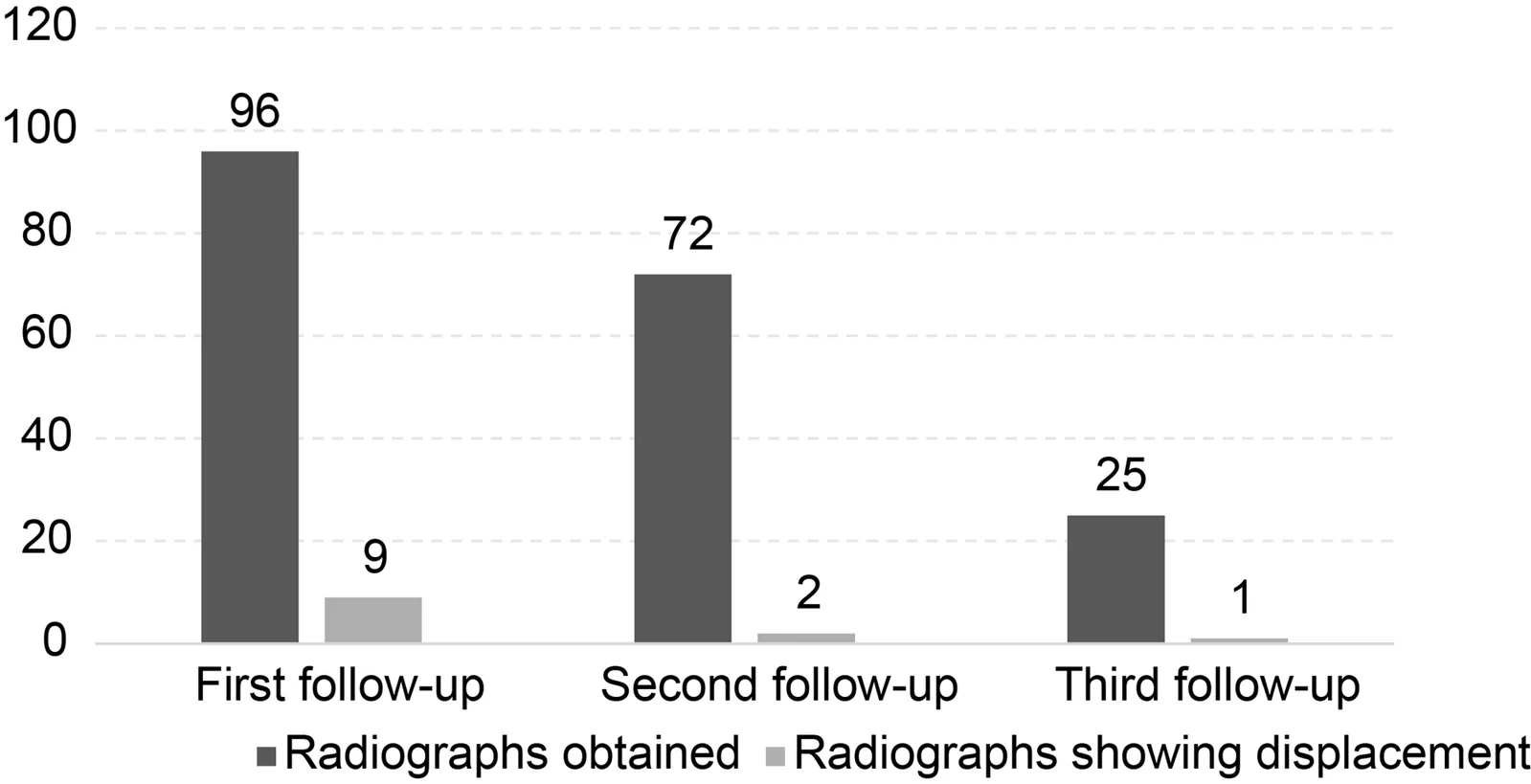
Number of follow-up radiographs and secondary displacements at each timepoint

Of the 96 patients who underwent radiography at the two-week follow-up, nine showed secondary fracture displacement (Fig. 5). Two demonstrated severe displacement; one subsequently had malunion and one had radiographic non-union.

#### Imaging data

**Fig. 6:**
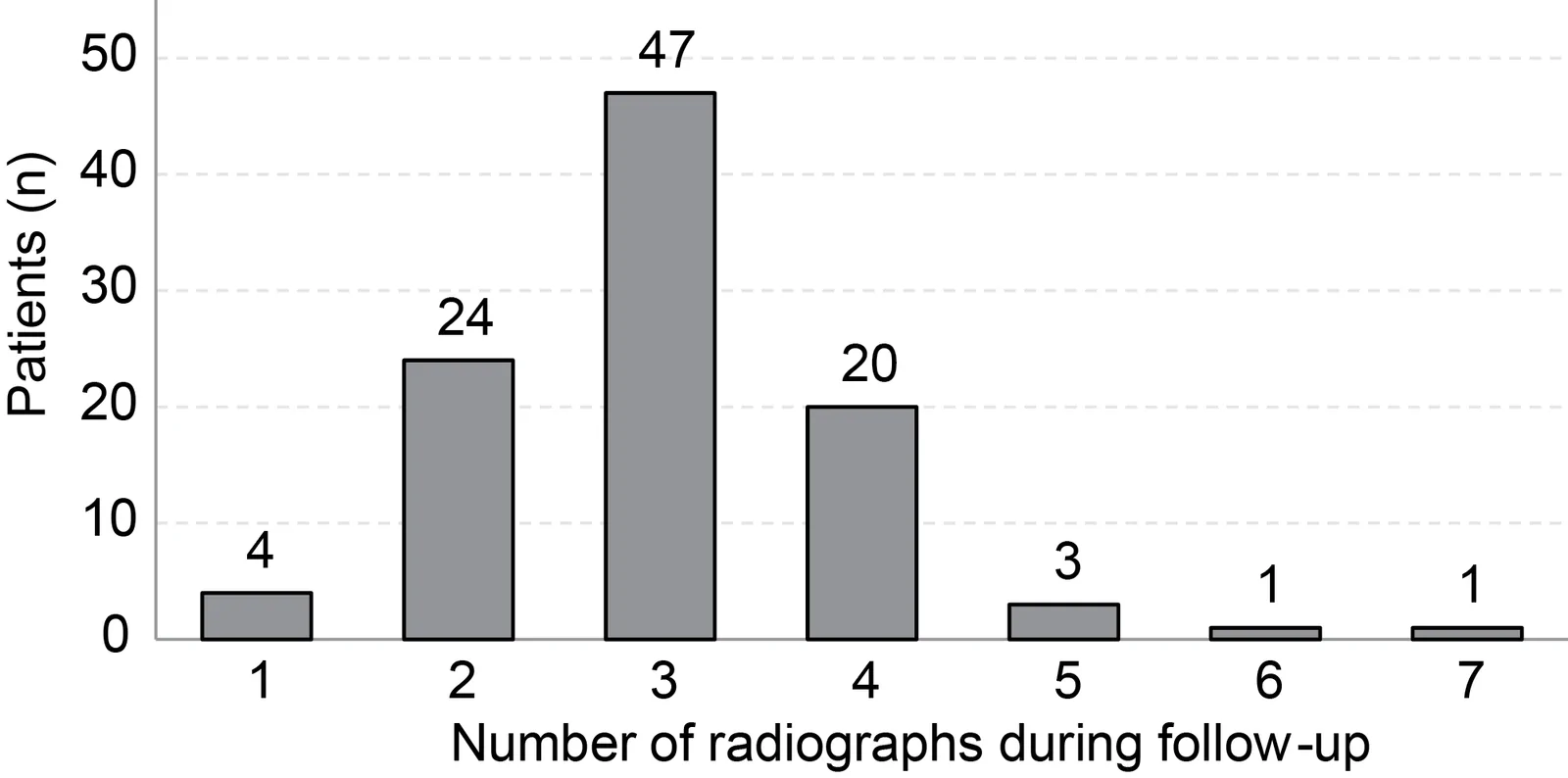
Total number of radiographs per patient during the observed treatment period (including the initial ED radiograph). Number of radiographs obtained per patient during follow-up

Patients underwent a mean of 3.0 radiographs (SD, 0.99; range, 1–7), including the initial ED radiograph (Fig. 6). Twenty patients underwent four radiographs in total. The indication for the additional radiograph was not systematically captured as a study variable, and the present data therefore cannot determine whether those examinations were clinically justified. Twenty-nine patients underwent CT imaging; one of these patients underwent two CT scans. In 24 patients, CT was performed during the initial ED assessment for fracture characterization or operative planning rather than as routine follow-up imaging. One patient underwent MRI and five underwent shoulder ultrasonography, three of which demonstrated rotator cuff pathology.

#### Observed treatment period

The observed treatment period, defined as the interval from the initial ED visit to the final outpatient clinic visit, lasted a median of 59 days (IQR, 59). Patients treated conservatively had a shorter observed treatment period (median, 58 days; IQR, 22) than those who underwent surgery (median, 155 days; IQR, 261), reflecting longer-term postoperative follow-up in some patients.

## Discussion

Routine follow-up radiographs had an observed management yield of 0% in this consecutive cohort. This result is clinically informative because it quantifies how infrequently scheduled imaging was associated with an actual treatment-plan change in this setting. It should not be interpreted as proof that routine radiographs can be omitted safely or that other surgeons would have made identical decisions. The finding is directionally consistent with previous studies reporting a low frequency of treatment change after follow-up imaging [9, 12]. Barreto Rocha et al. [9] reported conversion from conservative to operative treatment in a minority of older patients, including late conversions related to non-union or post-traumatic arthritis. Jacxsens et al. [12] concluded that serial radiological evaluation may be unnecessary for many one-part proximal humerus fractures, except when comminution is present.

The additional imaging observed in this cohort should be interpreted cautiously. Twenty patients underwent four radiographs in total, mainly due to requests from patients due to ongoing discomfort. CT imaging was predominantly obtained during the initial ED assessment for fracture characterization or operative planning and is conceptually distinct from universally scheduled outpatient radiographs. A future selective-imaging protocol should be evaluated prospectively and should preserve clinician-directed imaging when symptoms, function, fracture morphology, postoperative findings, or uncertainty about healing create a specific clinical indication.

### Limitations

This study has several limitations. First, the defined four-month sampling frame yielded 100 patients and no formal sample-size calculation was performed. A larger multicentre cohort would improve precision and permit analyses within clinically important fracture subgroups.

Second, the primary outcome reflected actual documented management within one hospital organisation. No independent blinded panel assessed whether other surgeons would have recommended a different strategy, including for the nine patients with secondary displacement. Institutional treatment preferences, observer bias, and confirmation bias may therefore have influenced the observed management yield and limit external validity. Reframing the endpoint as observed treatment-plan change, rather than the intrinsic necessity of imaging, is essential to interpreting these data.

Third, functional outcomes, pain, patient reassurance, and other potential benefits or harms of imaging were not measured. Follow-up extended to the final documented outpatient clinic visit, but later events after discharge may not have been captured. The present data therefore cannot establish that omitting scheduled radiographs is safe or that patient outcomes are unaffected. In addition, the cohort included few three- or four-part fractures and only 13 surgically treated patients; the findings should not be generalized to complex, severely displaced, or postoperative fractures.

Fourth, fracture morphology was summarized using the Neer classification, which has limited inter- and intra-rater reliability [13], and fracture location was not analysed in greater detail. The study period also overlapped with the COVID-19 pandemic, which may have influenced attendance, follow-up patterns, and treatment thresholds. Finally, the indication for additional radiographs beyond the routine schedule was not systematically captured, preventing assessment of whether these examinations were clinically justified.

Radiographs were assessed in routine clinical practice by multiple clinicians within the same hospital organisation, without independent blinded radiographic adjudication. This design reflects actual care but may introduce observer, confirmation, and institutional bias.

### Strengths

This study benefits from a clearly defined consecutive cohort managed under a stable institutional follow-up routine. Review of all available radiographs and clinic notes until the final outpatient visit allowed the primary outcome to reflect documented decisions across the full observed treatment period. Reporting the exact confidence interval provides a transparent measure of statistical precision and distinguishes a low observed management yield from proof of no benefit.

## Conclusion

In this consecutive single-centre cohort, scheduled follow-up radiographs were not associated with a documented treatment-plan change; the observed management yield was 0. This finding is clinically informative but does not establish that routine radiographs can be omitted safely or that management would be identical across surgeons. It supports a larger multicentre study incorporating independent radiographic adjudication, functional outcomes, and predefined clinical triggers to determine whether selective imaging can safely replace universal scheduled radiography in uncomplicated, clinically improving proximal humerus fractures.

## Data Availability

The datasets generated and analyzed during the current study are not publicly available due to patient privacy restrictions but are available from the corresponding author on reasonable request.
